# Friction and Wear Behavior of Alumina Composites with In-Situ Formation of Aluminum Borate and Boron Nitride

**DOI:** 10.3390/ma13204502

**Published:** 2020-10-11

**Authors:** Ashish K. Kasar, Pradeep L. Menezes

**Affiliations:** Department of Mechanical Engineering, University of Nevada, Reno, NV 89501, USA; akasar@nevada.unr.edu

**Keywords:** sliding test, wear rate, friction, hardness, composite

## Abstract

Wear and friction properties of Al_2_O_3_ composite reinforced with in-situ formed aluminum borate (9Al_2_O_3_·2B_2_O_3_) and hexa-boron nitride (h-BN) have been investigated. The initial constituents for the composites were Al_2_O_3_, AlN, and H_3_BO_3_. The H_3_BO_3_ was used as a source of B_2_O_3_, where B_2_O_3_ reacted with AlN and Al_2_O_3_ to form in-situ h-BN and 9Al_2_O_3_·2B_2_O_3_. Based on the thermodynamic calculation and phase transformation, four different compositions were selected. First, the powders were mixed by ball milling followed by compaction at 10 MPa. The compacted pellets were sintered at 1400 °C in vacuum. The composites were characterized using X-ray diffraction followed by hardness measurement and reciprocating sliding test against alumina and steel balls. The X-ray diffraction results revealed the formation of in situ phases of 9Al_2_O_3_·2B_2_O_3_ and h-BN that improved the tribological properties. By comparing the tribological performance of different composites, it was found that the hard 9Al_2_O_3_·2B_2_O_3_ phase maintains the wear resistance of composites, whereas the coefficient of friction is highly dependent on the counter ball. Against alumina ball, the lowest coefficient of friction was observed for the composites with maximum h-BN concentration and minimum aluminum borate concentration, whereas the opposite trend was observed against the steel ball.

## 1. Introduction

Ceramic materials are potential candidates for wear-resistance components because of their hardness, corrosion resistance, and high-temperature stability. However, their tribological applications are restricted by their poor lubricating property. To overcome the poor lubricity, solid lubricants, such as graphite [1,2], graphene [3,4], MoS_2_ [5], and CaF_2_ [6], are used in ceramic matrixes to form a self-lubricating ceramic composite with improved tribological properties. The addition of solid lubricants leads to the formation of a lubricative layer at the interface during sliding and reduces friction [7,8].

Alumina is one of the most employed ceramic materials, showing an excellent combination of properties that includes a high melting point, hardness, and corrosion resistance. Alumina also possesses good tribological properties, and with the addition of solid lubricants, the tribological properties have been improved [1,2,3,4,5,6,9]. Hexagonal boron nitride (h-BN) is one of the potential solid lubricants that has been used for synthesizing self-lubricating composites. However, h-BN easily oxidizes or hydrolyses [10,11]. Therefore, it is suitable for non-oxidative, vacuum, and inert atmospheres, even up to high temperatures. The effect of h-BN has been investigated in various ceramic matrices for mechanical and tribological performance. For example, Skopp et al. [12] studied the effect of h-BN content in the Si_3_N_4_ matrix on the tribological properties. The composites were prepared by hot isostatic pressing. The lower coefficient of friction (lesser than 0.2) of self-mated Si_3_N_4_-20BN composite was observed at room temperature for sliding velocities between 0.03 to 3.5 m/s at a normal load of 10 N, whereas the wear coefficient (K_v_, mm^3^/N.m) was reduced by a factor of 10. However, the K_v_ was still in the range of 10^−4^ to 10^−3^ mm^3^/N.m. The same group also performed the high temperature tests and found an increase in K_v_ up to 10^−2^ at 800 °C [13]. The authors also found that the mechanical properties were deteriorated by the addition of h-BN in the Si_3_N_4_ matrix.

Similarly, Wei et al. [14] studied the effect of h-BN in the Si_3_N_4_-Y_2_O_3_-Al_2_O_3_ matrix. The mass ratio of the matrix Si_3_N_4_-Y_2_O_3_-Al_2_O_3_ was 94:4.5:1.5, and h-BN content varied from 0 to 10 vol.%. Similar to the previous study, the mechanical property in terms of Vickers hardness was reduced from 14.5 to 10.9 GPa with the addition of 10 vol.%. The tribological test at 4 N against Si_3_N_4_ showed a slight decrease in friction coefficient from ~0.8 to ~0.7 with an addition of 10 vol.%. However, in this work, the wear was not quantified. In another work, h-BN was used in yittria stabilized zirconia (YSZ) coating fabricated by thermal spray coating method [15]. A pin-on-disk test was carried out against the 100Cr6 steel ball at 5 N normal load. The friction coefficient with the addition of 5 wt.% h-BN reduced from 0.55 to ~0.43, and K_v_ was observed in the range of 5 × 10^−5^ to 10 × 10^−5^ mm^3^/N.m. Based on the wear mechanism, it was suggested that under the tested parameters, the coating with 2.5 wt.% h-BN provided a smooth wear track by the formation of a lubricating film and yielded the lowest K_v_. The lesser h-BN provided wear track with groove formation and higher h-BN content observed with delamination. In these both conditions, the wear rate was higher.

Moreover, the direct addition of h-BN particles causes agglomeration and preexisting platelets that can further create complexities to achieve uniform distribution of h-BN [16,17]. It has also been observed that the direct addition of h-BN in various matrices inhibits sintering [18,19]. To overcome these issues, researchers have used the in-situ synthesis technique to produce BN in the SiC [20]–Si_3_N_4_ [21] ceramic matrix. In-situ BN has also been produced in Al_2_O_3_ matrix by using B_2_O_3_ and AlN to improve fracture toughness [22] and machinability [23], and to understand the reaction kinetics of in-situ formation [24]. However, the tribological properties of in situ BN-Al_2_O_3_ have not been studied.

The stability of the formed solid lubricant layers at the interface during sliding relies on the hardness of the matrix or any secondary additives [25,26]. In general, solid lubricant materials are soft in nature. Therefore, hard matrix or secondary materials are suitable to stabilize the solid lubricant layer at the interface and avoid further wear. For example, Ran et al. [27] studied the nano-hardness variation on the wear track and outside of the wear track for CuO-Yttria-doped Zirconia composites. The lower hardness values on the wear track (6 GPa), compared to higher hardness value (14 GPa) outside of the wear track, suggest that the lubricating layer, formed on the wear track during sliding, is significantly softer than the overall composite material. Moreover, it is clear that the soft layer was supported on a hard substrate.

**Figure 1:**
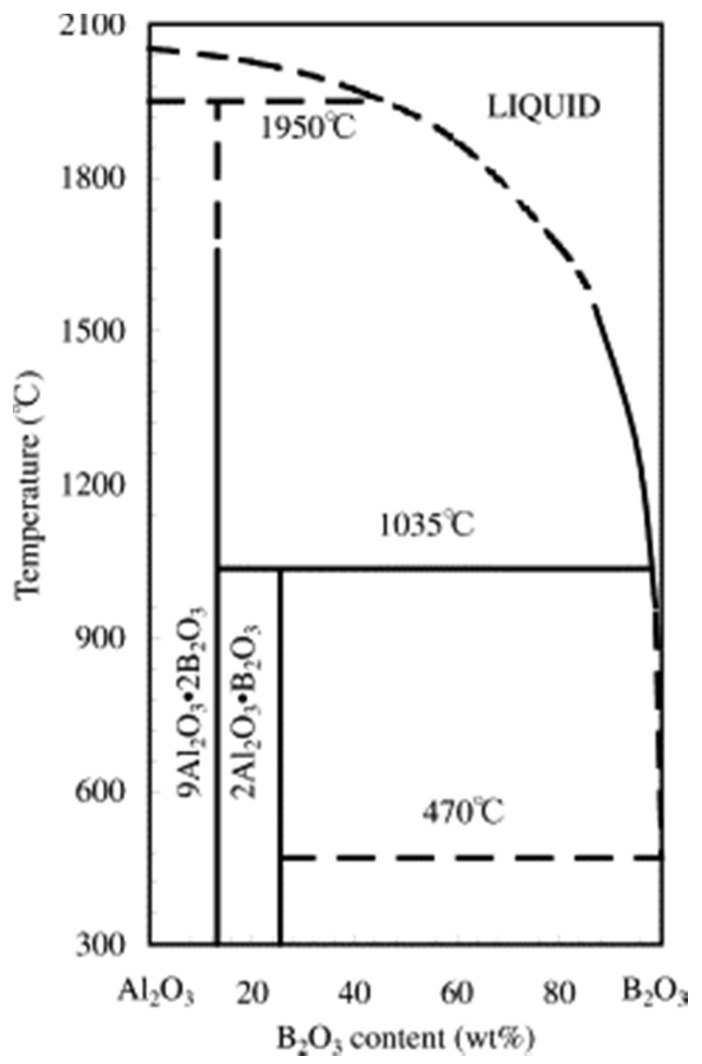
B_2_O_3_-Al_2_O_3_ phase diagram [31].

Similarly, in the current study, alumina acts as a hard substrate. In addition to alumina, this system utilized the in-situ chemical formation of hard aluminum borate phase (9Al_2_O_3_·2B_2_O_3_), as shown in the phase diagram of B_2_O_3_-Al_2_O_3_ in Figure 1. These aluminum borate phases are well known for hardness (7 in Mohs’ scale) [28]. Additionally, the addition of B_2_O_3_ has been observed to improve tribological performance by the formation of boric acid (H_3_BO_3_) [29,30]. In this work, we selected four different compositions based on a thermodynamic calculation to understand the effect of in-situ-formed h-BN and aluminum borate on the wear and friction properties.

## 2. Thermodynamics Consideration

Due to the melting of B_2_O_3_ at a temperature of 470 °C (Figure 1) and its evaporation, the reaction equations can be written as

(1) $$B_{2}O_{3}\left(l\right)+2\mathrm{AlN}={\;\mathrm{Al}}_{2}O_{3}+2\mathrm{BN}$$

(2) $$B_{2}O_{3}\left(g\right)+2\mathrm{AlN}={\;\mathrm{Al}}_{2}O_{3}+2\mathrm{BN}$$

The values of Gibbs free energy change of the Equations (1) and (2) at the standard condition in the temperature range of 527–1927 °C are given by [31]

(3) $$\Delta G_{T}{}^{o}\left(1\right)=\;\Delta H_{298}{}^{o}-T\Delta S^{o}=-288193+54.19T\;\left(J\right)$$

(4) $$\Delta G_{T}{}^{o}\left(2\right)=\;\Delta H_{298}{}^{o}-T\Delta S^{o}=-705589+226.07T\;\left(J\right)$$

where T is the temperature in Kelvin. These data suggest exothermic reaction, because they have large negative values of $\Delta G_{T}{}^{o}\left(1\right)$ and $\Delta G_{T}{}^{o}\left(2\right)$ that suggest Equations (1) and (2) will occur thermodynamically under standard condition.

In this work, boric acid (H_3_BO_3_) was used as a source of B_2_O_3_, and thermodynamic studies suggest that complete dehydration of boric acid, as shown in Equation (5), can be achieved between 130 and 330 °C with a heating rate of 5 °C/min [32].

(5) $$H_{3}{\mathrm{BO}}_{3}\left(s\right)=\;1/2B_{2}O_{3}\left(s\right)+3/2H_{2}O\;\left(g\right)$$

## 3. Materials and Methods

### 3.1. Sample Preparation

Al_2_O_3_ (mean particle size D50 = 20 μm, AdValue Technology, Tucson, AZ, USA), AlN (particle size 4 μm, Beantown Chemical, Hudson, NH, USA), and boric acid (Ward’s science, Rochester, NY, USA) were used in this study. The Vickers hardness of pure initial constitutes, i.e., Al_2_O_3_, AlN, and boric acid, are 1800, 1300, and 153, respectively [33,34]. The stoichiometric amount of powders was taken based on the reaction Equations (1), (2) and (5). Four compositions were selected to understand the importance of aluminum borate and h-BN in alumina matrix to investigate wear and friction properties. The compositions are listed in Table 1.

The amount of boric acid was calculated by considering the molecular weight of B_2_O_3_ (69.62 g/mol) and H_3_BO_3_ (61.83 g/mol) to achieve the desired B_2_O_3_ in the matrix. For composition A10 and A20, the number of moles of AlN are twice that of B_2_O_3_ to achieve Equations (1) and (2), and only h-BN formation was expected, whereas compositions B10 and B20 were expected to form aluminum borate along with h-BN due to the presence of excess B_2_O_3_. In the Table 1, the resulted h-BN concentration was calculated based on Equations (1) and (2), which suggests the number of AlN moles will be equal to number of h-BN moles. The number of moles per 100 g were converted into wt.%, yielding 7.11 wt.% h-BN in the A10 and B10 composites, whereas the A20 and B20 composites carried 14.25 wt.% h-BN. The powders were thoroughly mixed using ball milling (Across international, Reno, NV, USA). To avoid any contamination, the powders were ball-milled using alumina jar and alumina balls for 30 min with a rotating speed of 300 rpm. Cylindrical green pellets of 20 mm diameter were produced by compacting the mixed powders under 10 MPa for 5 min at room temperature. These pellets were sintered at 1400 °C in a vacuum for 1 h with a heating and cooling rate of 5 °C/min. After sintering, the density of the pellets was measured. For comparison, a pure alumina sample was prepared by the same methodology.

### 3.2. Characterization and Testing

Phase identification was carried out by X-ray diffraction (XRD) using Bruker-D2 phaser (Bruker, Madison, WI, USA) for a wide range of angles (2θ) ranging from 10° to 90°, with a scan speed of 0.0101. Micro-hardness of the composites was tested using Wilson Tukon^TM^ 1202 hardness tester (Buehler, Lake Bluff, IL, USA). The hardness tests were conducted using a load of 200 g with a dwell time of 10 s. At least 10 measurements were taken to calculate the average hardness of the composites.

Dry reciprocating linear sliding tests were performed on the composites using Rtec multi-function Tribometer (Rtec-instruments, San Jose, CA, USA). Alumina ball (900 HV) (McMaster-Carr, Atlanta, GA, USA) and 52,100 steel balls (740 HV) (McMaster-Carr, Atlanta, GA, USA) of 6.35 mm diameter were used as counterparts. The reciprocating tests were conducted using a track length of 10 mm for 20 cycles under 10 N normal load with a sliding speed of 1 mm/s. After the reciprocating sliding tests, the generated wear track was analyzed by an optical microscope and scanning electron microscope (SEM; JEOL JSM-6010LA, Peabody, MA, USA). The chemical characterization of the wear track was characterized by an energy dispersive spectrometer (EDS, JOEL, Peabody, MA, USA) equipped with the SEM.

## 4. Results and Discussion

### 4.1. Phase Identification Using X-ray Diffraction

**Figure 2:**
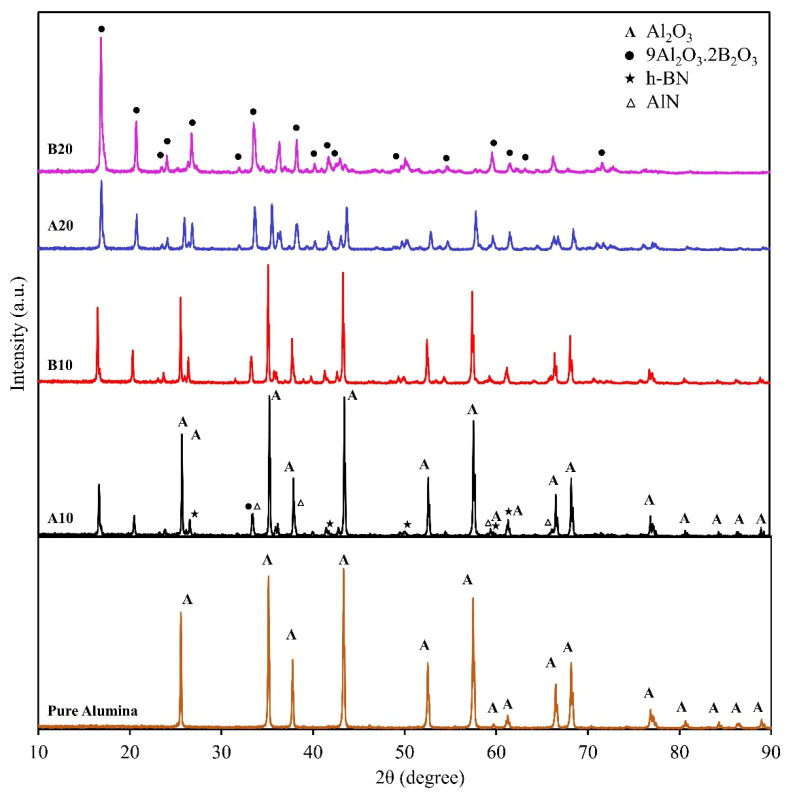
XRD spectrum of the pure alumina sample and the composites (A10, B10, A20, and B20).

The XRD peaks were identified using the crystallography open database (COD) [35]. All the peaks were identified, suggesting that the phases are Al_2_O_3_ (COD 9007634), 9Al_2_O_3_·2B_2_O_3_ (COD 9005085, aluminum borate), h-BN (COD 5910079), and AlN (COD 1010514), as shown in Figure 2. The peaks are only denoted by symbols in pure alumina sample and A10 and B20 spectrum, to avoid the clustering of symbols. The Al_2_O_3_, h-BN, and AlN peaks are denoted in A10 composite spectrum, whereas aluminum borate peaks are highlighted in B20 composite spectrum. The same peaks can be seen in all the spectrums with changes in intensities. For example, the peaks of Al_2_O_3_ can be clearly seen in A10 composite, and its peak intensity decreases with the addition of B_2_O_3_ due to the formation of aluminum borate. These XRD peaks suggest that the B20 composite will have the highest aluminum borate concentration. Figure 2 also confirms the formation of h-BN, as the h-BN peaks can be clearly observed in A10, B10, and A20 composites. In B20 composite, h-BN peaks are suppressed to a high amount of aluminum borate. Small intensity peaks of AlN were also observed that suggest the presence of unreacted AlN in the matrix.

### 4.2. Densification

The lower density of the sintered pellets was observed in the range of 1.3 to 1.59 g/cm^3^. Theoretical and sintered densities of all the four compositions are mentioned in Table 2. Theoretical density was calculated based on the weight fraction of Al_2_O_3_, B_2_O_3_, and AlN, which are higher than the sintered density. Such lower density is due mainly to the dehydration of boric acid, which is the source of B_2_O_3_ in the composites. As the boric acid/B_2_O_3_ content increased in the composite, the sintered density decreased. The formation of aluminum borate during sintering is also responsible for lower density, because aluminum borates are known for their lower density (2.9 g/cm^3^), compared to Al_2_O_3_ (3.95 g/cm^3^) [20]. From Table 2, it can also be seen that with increasing B_2_O_3_ content in the matrix (comparing B10 with A10 and B20 with A20), the relative density has decreased due to the formation of aluminum borate. It has also been suggested that h-BN containing composites shows poor densification with higher h-BN content due to the formation of h-BN flakes with different basal plane orientation and thus lowers the densification [36,37].

### 4.3. Hardness

**Figure 3:**
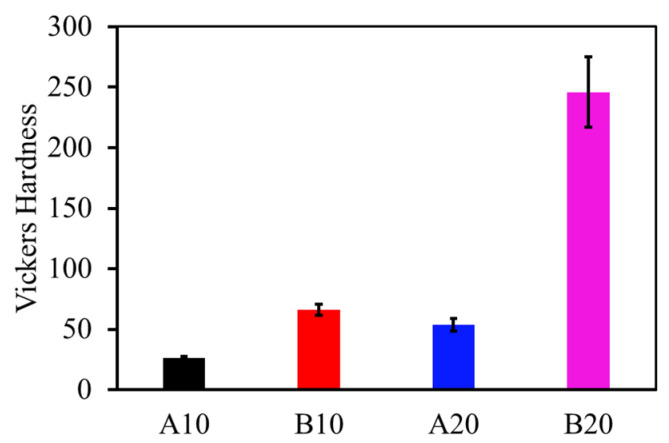
Vickers hardness of the composites.

The Vickers hardness values of the composites are plotted in Figure 3. The effect of different phases can be clearly observed in the hardness values. For example, B10 has higher hardness than A10. Similarly, B20 has higher hardness than A20. This behavior is due to the amount of aluminum borate phase in the matrix, because aluminum borate has a higher hardness. The maximum amount of aluminum borate in composite B20 results in the maximum hardness of 246 ± 29 HV. The hardness of the pure alumina sample was observed to be 28 ± 6 HV, which is close to the hardness of the A10 composites.

### 4.4. Tribological Results

**Figure 4:**
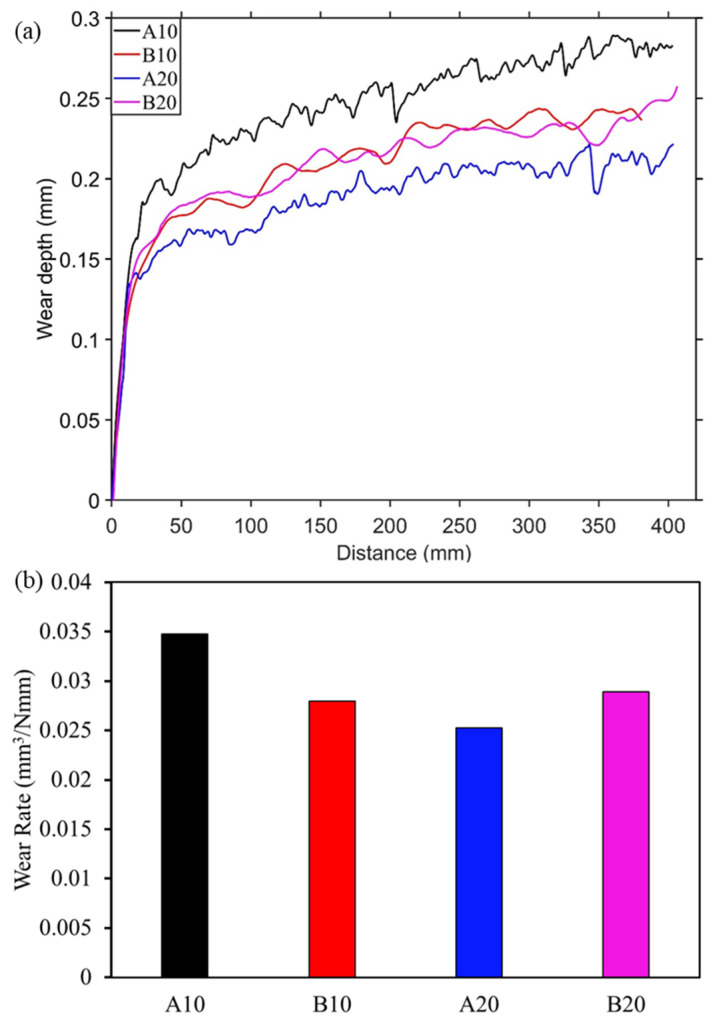
Wear measurement against alumina ball. (**a**) Wear depth during sliding and (**b**) final wear rate.

**Figure 5:**
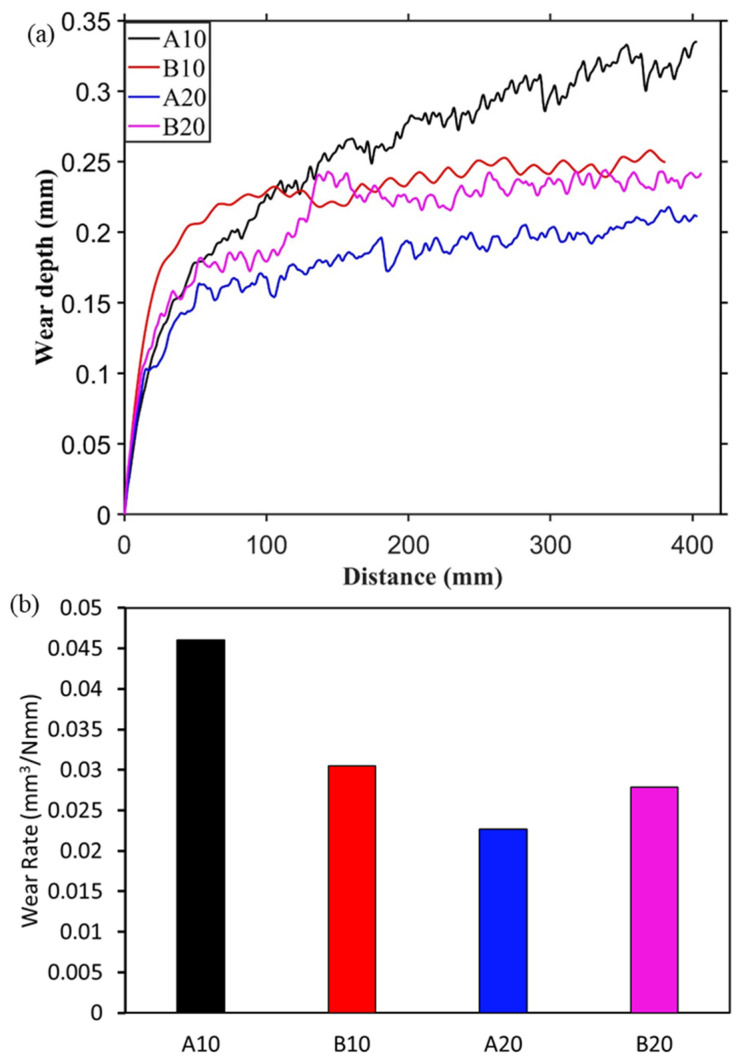
Wear measurement against steel ball. (**a**) Wear depth during sliding and (**b**) final wear rate.

During the reciprocating sliding test against alumina and E51200 steel ball, wear depth was recorded and plotted in Figure 4a and Figure 5a, respectively. Initially, a steep increase in wear depth can be seen for all four composites against both the counter materials, followed by gradual or stable wear depth. This behavior is typical self-lubricating material behavior [38], where initial wear causes the formation of a lubricating layer at the interface that stabilizes and reduces further wear. Moreover, the A10 composite shows a continuous increase in wear depth compared to other composites.

After the 20 cycles of reciprocating sliding, the final wear rate was calculated and plotted in Figure 4b and Figure 5b. The specific wear rate was calculated based on the recorded wear depth against the spherical ball. The wear rate against the steel ball was slightly higher than against the alumina ball. In both Figure 4b and Figure 5b, it can be seen that the wear rate is decreasing from A10 composite to A20 composite then increasing for B20 composites. This behavior is consistent with both the counter materials, i.e., alumina and steel. This is due to an increased amount of harder aluminum borate phase from A10 to B20 composites, which provides a wear-resistance substrate to the solid lubricant h-BN. However, the increase in h-BN content from A20 to B20 composites led to an increase in wear rate. The wear rate of pure alumina sample against alumina and steel balls are 0.036 and 0.057 mm^3^/Nmm, respectively. In Figure 4b, the lowest wear rate is observed for the A20 composite, which is 31.5% lesser than the pure alumina sample. Similarly, against steel ball, the lower wear rate is observed for A20 (0.022 mm^3^/Nmm), which is ~60% less than the pure alumina sample.

**Figure 6:**
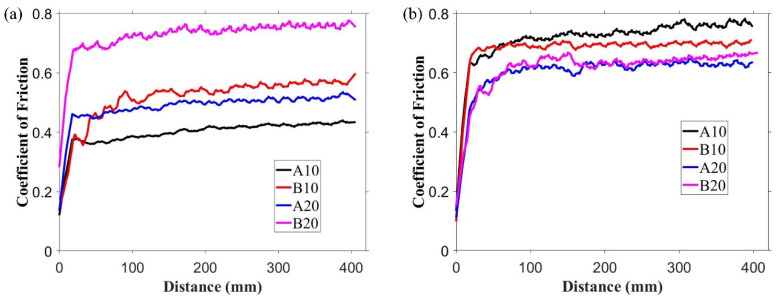
Coefficient of friction (COF) variation over the sliding distance against (**a**) alumina ball and (**b**) steel ball.

The observed coefficient of friction (COF) variations over the distance against alumina ball and steel ball are shown in Figure 6a,b, respectively. Against alumina ball, the lowest COF at the end of the test was observed for A10 composite (that is, 0.42); COF increased gradually, and the highest COF of 0.75 was observed for B20 composite. This COF trend is related to the phases present in the composites. As shown in XRD results (Figure 2), h-BN was present in the composite, which is a solid lubricant material used to lower the COF. However, the presence of aluminum borate lowered the h-BN amount in the matrix. This relative amount of h-BN and aluminum borate suggests the observed trend in COF against alumina ball. The average COF for pure alumina sample against alumina ball and steel ball was 0.83 and 0.81, respectively, which was higher than all the composites.

**Figure 7:**
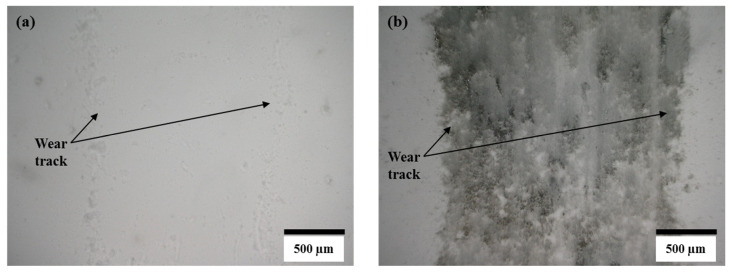
Optical micrographs of wear track on A10 composite after reciprocating sliding test against (**a**) alumina ball and (**b**) steel ball.

**Figure 8:**
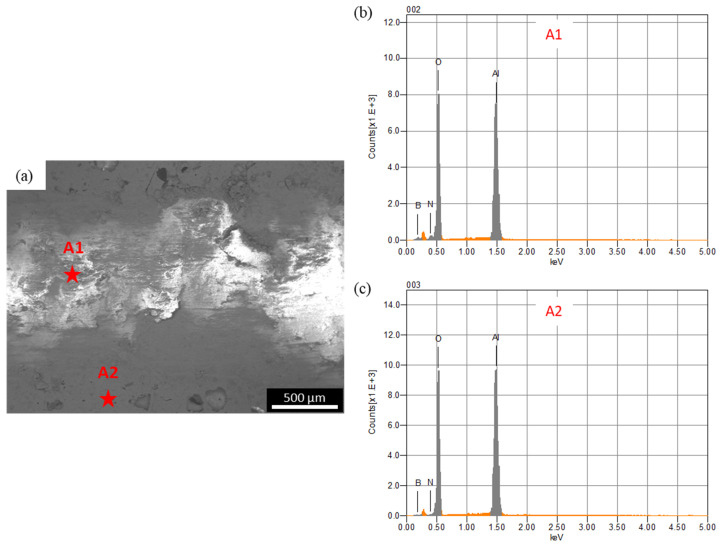
(**a**) SEM micrograph of the wear track on B20 composite against alumina ball; (**b**,**c**) are the EDS spectrum at A1 and At2, respectively.

**Figure 9:**
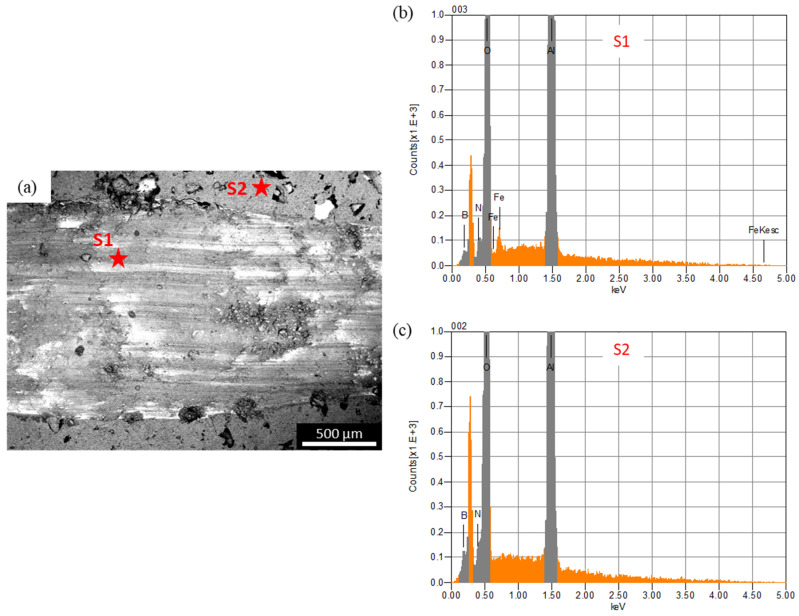
(**a**) SEM micrograph of the wear track on B20 composite against steel ball; (**b**) and (**c**) are the EDS spectrum at S1 and S2, respectively.

In the case of the steel ball, the friction was higher than the alumina ball, and an opposite trend was observed, compared to the COF trend observed against the alumina ball. This was because the steel ball had a lower hardness than the alumina ball. Moreover, during sliding, the steel ball wore out and transferred to the composite surface, as shown in the optical image of wear track of A10 composites (Figure 7b). The wearing out behavior of steel ball was observed against pure alumina sample and all four composites, which was further confirmed by EDS analysis of the wear track. SEM micrograph and EDS spectrums of the wear tracks on the B20 composite against alumina and steel ball are shown in Figure 8 and Figure 9, respectively. The EDS spectrum at S1 point on wear track against steel ball confirms the presence of Fe (Figure 9b). The EDS spectrum are also taken outside of the wear track (S2) that shows no Fe, confirming that the source of Fe is the counter steel ball. The EDS spectrum on (A1) and outside (A2) of the wear track generated against alumina shows the expected elements, which are Al, O, B, and N.

Against the steel ball, the transfer layer of steel on the composites changed the interacting material pair at the interface, i.e., the interaction between steel against steel materials occurred instead of alumina composite against steel. This, in turn, changed the friction behavior at the interface. For example, A10 composite showed the highest COF (Figure 6b) because the transfer of steel on the alumina composite hinders the formation of h-BN lubricating layer. Therefore, the formation of the steel transfer layer was the main reason for the higher COF of the composites against steel ball, compared to observed COF against alumina ball.

In addition, previous studies suggest that the tribo-oxidation of the steel occurs during sliding [39,40,41]. The tribo-oxidation of steel can also be one of the reasons behind the observed friction trend. However, the use of alumina ball eliminates the impact of tribo-oxidation and allow to focus on the properties of the composites.

## 5. Conclusions

The low-density alumina-based composite was successfully synthesized with the in-situ formation of aluminum borate and h-BN phase. It was observed that the formation of aluminum borate helps to achieve a lower density of the composite and also reduces the wear rate due to its hardness. A20 composite with the maximum amount of aluminum borate was observed to have the lowest wear rate. On the other hand, maximum COF was observed for B20 composite against alumina ball due to a lesser amount of h-BN. Against alumina ball, optimum wear and friction behavior were observed for A20 composite with COF of 0.5 and a specific wear rate of 0.024 mm^3^/Nmm. However, against steel ball, B20 composite had the minimum COF, whereas A20 composite resulted in the highest COF, due to the transfer layer of steel on the composite that changed the material interaction pair at the interface, which thus changed the frictional behavior. A similar wear rate trend was observed in the case of alumina counter material. This study suggests that the amount of in situ formed aluminum borate and h-BN should be controlled as per the counter material for optimum friction and wear properties.

## Figures and Tables

**Table 1:** Compositions of composites with varying amounts of B_2_O_3_ and AlN that resulted in hexa-boron nitride (h-BN) formation.

Composite Designation	Wt.%			
	B_2_O_3_	AlN	Al_2_O_3_	Resulted h-BN
A10	10	11.75	78.25	7.11
B10	20	11.75	68.25	7.11
A20	20	23.55	56.45	14.25
B20	40	23.55	36.45	14.25

**Table 2:** Theoretical and sintered density of the composites.

Composite	Density (gm/cm3)	
	Theoretical	Sintered
A10	3.65	1.59
B10	4.03	1.43
A20	3.40	1.47
B20	3.11	1.3
